# Structural connectome constrained graphical lasso for MEG partial coherence

**DOI:** 10.1162/netn_a_00267

**Published:** 2022-10-01

**Authors:** Anirudh Wodeyar, Ramesh Srinivasan

**Affiliations:** Department of Cognitive Sciences, University of California, Irvine, California, USA; Department of Statistics, University of California, Irvine, California, USA; Department of Biomedical Engineering, University of California, Irvine, California, USA; Department of Mathematics and Statistics, Boston University, Boston, Massachusetts, USA

**Keywords:** MEG, Gaussian graphical model, Coherence, Structural connectivity, Functional connectivity

## Abstract

Structural connectivity provides the backbone for communication between neural populations. Since axonal transmission occurs on a millisecond time scale, measures of M/EEG functional connectivity sensitive to phase synchronization, such as coherence, are expected to reflect structural connectivity. We develop a model of MEG functional connectivity whose edges are constrained by the structural connectome. The edge strengths are defined by partial coherence, a measure of conditional dependence. We build a new method—the adaptive graphical lasso (AGL)—to fit the partial coherence to perform inference on the hypothesis that the structural connectome is reflected in MEG functional connectivity. In simulations, we demonstrate that the structural connectivity’s influence on the partial coherence can be inferred using the AGL. Further, we show that fitting the partial coherence is superior to alternative methods at recovering the structural connectome, even after the source localization estimates required to map MEG from sensors to the cortex. Finally, we show how partial coherence can be used to explore how distinct parts of the structural connectome contribute to MEG functional connectivity in different frequency bands. Partial coherence offers better estimates of the strength of direct functional connections and consequently a potentially better estimate of network structure.

## INTRODUCTION

Electrophysiological signals are sampled on a millisecond time scale capturing aggregate synaptic activity from populations of neurons. These neuro-physiological signals have intrinsic time scales, organized in frequency bands; and intrinsic spatial organization, organized by functional localization and integrated by the anatomical connectivity (Nunez & Srinivasan, 2006). Functional connectivity (FC) (Friston, 2011) refers to statistical dependence between signals recorded from two different areas of the brain, usually measured in a predefined frequency band. This broad definition encompasses different preprocessing methods and statistical models that emphasize different temporal and spatial scales of the underlying brain activity.

Coherence is a widely used measure of electroencephalography and magnetoencephalography (M/EEG) functional connectivity (Nunez & Srinivasan, 2006). Coherence is modulated across different cognitive tasks and clinical disease states (Baillet, 2017; Gross et al., 2013; Rouhinen, Panula, Palva, & Palva, 2013; Roux & Uhlhaas, 2014; Siebenhühner, Wang, Palva, & Palva, 2016; Stam, 2014). Coherence is expected to reflect delayed signal transmission along white-matter tracts, that is, structural connections (Abdelnour, Voss, & Raj, 2014; Chu et al., 2015; Meier et al., 2016; Nunez & Srinivasan, 2006; Schneider, Dann, Sheshadri, Scherberger, & Vinck, 2020; Srinivasan, Winter, Ding, & Nunez, 2007) and is thus used to characterize network structure.

However, mapping coherence to the anatomy is difficult due to its susceptibility to inflation from leakage effects. Leakage effects are the shared activity across brain sources caused by the limited resolution of source localization methods (Baillet, Mosher, & Leahy, 2001; Brookes et al., 2011; Gross et al., 2001; Hämäläinen & Ilmoniemi, 1994; Wipf & Nagarajan, 2009) to spatially separate source activity mixed by EEG volume conduction and MEG field spread (Nunez & Srinivasan, 2006; Srinivasan et al., 2007). Leakage effects result in common signals with zero phase difference between sources. One approach suggested to reduce leakage effects, the imaginary coherence (Nolte et al., 2004), is based on using only the projection of signals onto a phase difference of +/−90 degrees. However, this distorts the interpretation of the strength of functional connectivity, by weighting toward signals with preselected phase differences. Moreover, this approach is still susceptible to spurious connections when genuine long-range connections exist at a delay and this activity is leaked to neighboring regions (Palva et al., 2018).

We can use coherence or imaginary coherence to define the network edge weights, a critical first step for analyzing network structure, for example, using graph theoretical methods (De Vico Fallani, Richiardi, Chavez, & Achard, 2014; Maldjian, Davenport, & Whitlow, 2014; Niso et al., 2015; Schoonheim et al., 2013). However, both coherence and imaginary coherence reflect activity over single and multistep structural connectivity (Abdelnour et al., 2014; Chu et al., 2015; Meier et al., 2016). This distorts the definition of a path (Avena-Koenigsberger, Misic, & Sporns, 2018; Blinowska & Kaminski, 2013; Kaminski & Blinowska, 2018) over an undirected network and thus raises questions about the validity of network structure analyses using networks defined by the strength of coherence.

In contrast to the coherence or imaginary coherence, partial coherence accounts for both instantaneous and lagged shared information across multiple areas (Dahlhaus, 2000; Reid et al., 2019; Sanchez-Romero & Cole, 2021). Partial coherence has a long history in neuroscience: initially applied to spike trains (Rosenberg, Halliday, Breeze, & Conway, 1998) and generalized in Baccalá and Sameshima (2001) to the partial directed coherence. The real-valued analogue, partial correlation, has been applied to fMRI data across many studies (Hinne, Janssen, Heskes, & van Gerven, 2015; Huang et al., 2010; Ng, Varoquaux, Poline, & Thirion, 2012; Ryali, Chen, Supekar, & Menon, 2012; Smith et al., 2011; Varoquaux, Gramfort, Poline, & Thirion, 2010; Wodeyar, Cassidy, Cramer, & Srinivasan, 2020). Partial coherence represents the strength of linear relationships between a pair of brain areas when accounting for their relationships with all other brain areas (Dahlhaus, 2000; Epskamp & Fried, 2018; Whittaker, 2009). It reduces false positive detection of direct connections resulting from activity over indirect connections, as would result from leakage effects and multistep paths. Thus, we can better interpret partial coherence as connection strength to define a functional network. However, partial coherence estimation can be challenging. Most previous studies using partial coherence have focused on cases where there are only a few nodes in the network or used the L2-norm penalization for regularization (Baccalá & Sameshima, 2001; Colclough et al., 2016; Dahlhaus, 2000; Medkour, Walden, & Burgess, 2009; Ter Wal et al., 2018), without obvious justification. The use of the L2-norm is counterintuitive, as the structural connectivity of the brain is known to be sparse, and there is little reason to minimize the edge strengths. In the fMRI literature, when estimating partial correlation, several studies have experimented with alternative regularization approaches: L1-norm (Huang et al., 2010), elastic net (Ryali et al., 2012), group-based penalization approaches (Varoquaux et al., 2010), edge-specific penalization (Ng et al., 2012), as well as Bayesian approaches to estimation (Hinne et al., 2015). However, these alternative regularization approaches have not been attempted in partial coherence estimation, in part because of the difficulty in implementing them.

We expect that functional connectivity is constrained by the structural connectome. In this article, we make explicit use of the structural connectome to facilitate regularization of partial coherence estimates. We use a graphical lasso technique modified to use the structural connectome to guide the L1 penalization, a method we call the adaptive graphical lasso (AGL). To our knowledge, this is the first time that the graphical lasso (L1-norm), and further the graphical lasso using a constraint-based penalization, has been used to estimate partial coherence for neural signals (Colclough et al., 2016; Ter Wal et al., 2018). We select the lasso penalization through a novel cross-validation technique that separately identifies the optimal penalization on and off the structural connectome. If the penalization is lower for edges in the structural connectome, we have clearly identified that the pattern of connectivity is influenced by the structural connectome. Note that the entire structural connectome need not be estimated in the partial coherence, a subset may be estimated as a function of the data. Through simulations, we aim to demonstrate that (1) the partial coherence can be estimated accurately using the AGL, (2) we can directly test whether the structural connectome is a useful constraint in network identification, and (3) the partial coherence serves as a better functional connectivity metric than the coherence or imaginary coherence. Finally, we use the AGL-estimated partial coherence to demonstrate distinct contributions of the structural connectome to MEG signals in different frequency bands.

## METHODS

### Overview

This work is guided by the intuition that the statistics of neural activity data collected at the mesoscale (intracranial electrocorticography - ECoG) and macroscale (M/EEG) are constrained by structural connectivity of the axon fiber systems of the cortex. As such, we have built a minimal generative computational model, representing the partial coherence, that is derived from estimates of structural connectivity and we have developed a method to infer model parameters. We allowed the structural connectivity to potentially guide the estimation of the partial coherence and developed new simulations to link this work with M/EEG and ECoG data.

### Structural Connectome

We built a template of the structural connectome (SC) from a probabilistic atlas. We used streamlines generated with deterministic tractography by Yeh et al. (2018) using the HCP842 dataset (Van Essen et al., 2013) transformed to the MNI152 template brain obtained from the FMRIB Software Library (FSL). In this dataset experts vet the streamlines to remove potentially noisy estimates of axonal fibers. We applied the Lausanne parcellation (Cammoun et al., 2012) of 114 cortical and 15 subcortical regions of interest (ROIs) to the MNI152 template brain and generated a volumetric representation for each region of interest using the *easy_lausanne* toolbox (Cieslak, 2015). Each streamline was approximated by a single 100-point cubic spline using code adapted from the along-tract-stats toolbox (Colby et al., 2012). By identifying the streamlines which terminated in a pair of ROIs, we were able to create the SC for the Lausanne parcellation. Each streamline only connected a single pair of ROIs. An edge *W*_*ij*_ for ROIs *i* and *j* existed if there was a streamline connecting the pair.

**Figure 1.**
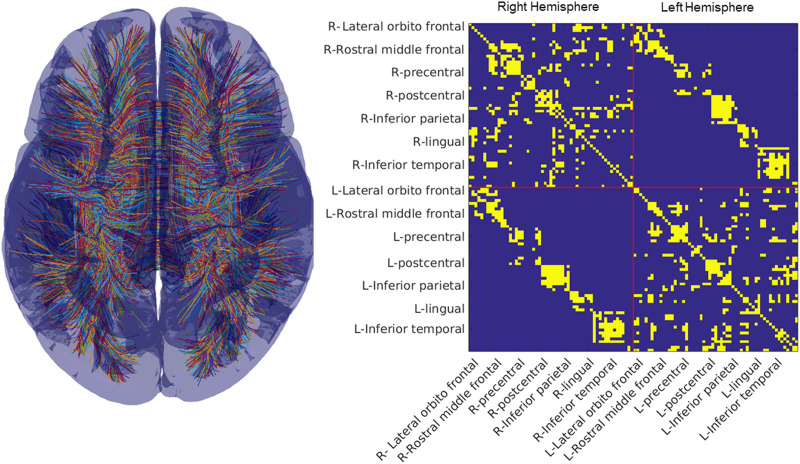
Structural connectome. (Left) We show streamlines derived from the work of Yeh et al. (2018) using the HCP-842 dataset. (Right) The structural connectome for the 114 areas of the Lausanne parcellation. We have labeled a subset of areas each with one to three subdivisions (see Cammoun et al., 2012, for all subdivisions of the Lausanne parcellation). We show the undirected and unweighted SC, with any nonzero edge being shown in yellow.

From this process, we built the 129 × 129 undirected and unweighted structural connectome with 1,132 edges. We reduced this matrix to 114 × 114 with 720 edges (see Figure 1) after removing all the subcortical structures and limiting interhemispheric connections to homologous white-matter tracts. This latter step helped remove potentially noisy estimates of connections (while potentially increasing false negatives) where streamlines intersected and passed outside the cortical surface before reaching the terminal point in a brain region. The resulting template of structural connectivity shown in Figure 1 is referred to as the structural connectome (SC). This template is incomplete in that it does not include subcortical to cortical projections. Thus, functional connectivity resulting from structural connections not captured by this template may exist in the data. Our estimation procedure for the graphical models of functional connectivity described in the next section allows for such connections, if needed, to account for the statistical structure in the data.

### Generative Model

#### Complex-valued Gaussian graphical model.

We assume that a vector of activity (**Z**) in one frequency band is a sample drawn from a complex-valued multivariate Gaussian. Here Φ is the precision—the unnormalized partial coherence—and is determined by the SC:

$$Z∼N\left(0,\Phi \right)$$ (1)

In the frequency domain, a signal can be characterized by samples of amplitude and phase, or equivalently, by complex-valued coefficients with real and imaginary parts corresponding to sine and cosine components of the signal.

The complex-valued multivariate Gaussian for a zero-mean (where *E*(**Z**) = 0 + 0*i*) process (Schreier & Scharf, 2010) is defined as:

$$\rho \left(Z\right)=\frac{1}{\pi^{n}\det^{\frac{1}{2}}\left(\Sigma \right)}\exp\left(-\frac{1}{2}{Z\Sigma }^{-1}Z^{H}\right)$$ (2)

where

$$\Sigma =\left[\begin{array}{cc} R_{zz} & {\tilde{R}}_{zz}\\ {\tilde{R}}_{zz}^{H} & {R_{zz}}^{H} \end{array}\right]$$ (3)

and

$$R_{zz}=E\left[{zz}^{H}\right];{\tilde{R}}_{zz}=E\left[{zz}^{T}\right]$$ (4)

The key parameter in this model is the covariance matrix **Θ** and its inverse, the *precision* matrix **Φ** = **Θ**^−1^. As defined in Equations 3 and 4, the covariance matrix for complex-valued data is composed of the familiar cross-spectrum **R**_**zz**_ and the complementary cross-spectrum $\tilde{R}$_**zz**_. Most spectral analysis methods only make use of **R**_**zz**_ and implicitly assume circular symmetry, that is, $\tilde{R}$_**zz**_ = 0 (Schreier & Scharf, 2010). In this case, the complex-valued data is labeled as *proper*. With the assumption of circular symmetry, we can parameterize the complex-valued Gaussian using the precision as:

$$\Phi ={{R_{zz}}^{-}}^{1}$$ (5)

Each value in the precision matrix **Φ** is the conditional covariance between any two variables (here, sources representing two ROIs) given the other variables (all other ROIs). The precision represents a model of functional connectivity—the *conditional dependence* between sources. The strength of the conditional dependence represents the linear relationship between any pair of sources when linear effects from all other sources are removed (see Section 2.2.2 of Pourahmadi, 2011 for an intuitive explanation in terms of multivariate linear regression). For any pair of sources, if the precision is zero, there is no need for a relationship between the sources to account for observed coherence. Such apparent coherences arise from connections mediated via other sources in the model. Note that the precision directly represents a complex-valued Gaussian graphical model (Whittaker, 2009).

In the generative model, we choose to set up the precision matrix **Φ** to have a nonzero entry only at edges that have a connection in the SC. We are assuming that in each frequency band, coherence represents the result of joint random fluctuations of a set of oscillators whose connections are determined by the SC. The precision vales are estimated using the graphical lasso in a cross-validated procedure that allows potentially using the SC as a guide for the L1 penalization. In this way the nonzero locations and values of the precision are determined by the data.

#### Adaptive graphical lasso.

The graphical lasso (Friedman, Hastie, & Tibshirani, 2008) is a method that has been applied in multiple fields in the past decade, from genomics (Menéndez, Kourmpetis, ter Braak, & van Eeuwijk, 2010) to fMRI functional connectivity (Ng et al., 2012; Ryali et al., 2012; Varoquaux et al., 2010; Wodeyar et al., 2020) and climate models (Zerenner, Friederichs, Lehnertz, & Hense, 2014). It is used to identify a sparse approximation to the regularized precision matrix while solving problems arising from rank deficiency and small numbers of samples. To apply the lasso, we optimize the penalized likelihood function for a multivariate Gaussian (Meinshausen & Bühlmann, 2006) to estimate the precision—where Θ (*R*_*zz*_ in Equation 4) is the cross-spectral density (CSD):

$$\hat{\Phi }=\arg\min_{\Phi ≻0}\left(-\log\left(\det\Phi \right)+\text{tr}\left(\Theta \Phi \right)+\lambda \sum_{j<k}\left|\Phi_{jk}\right|\right)$$ (6)

The penalization parameter λ in the graphical lasso determines the nonzero set of precision values. The output of the lasso from Equation 6 is the precision matrix $\hat{\Phi }$.

We made use of the lasso to estimate the precision while taking advantage of the knowledge of the SC to hypothesize the likely locations of nonzero precision values. We made use of the lasso optimization from quadratic approximation for sparse inverse covariance or QUIC (Hsieh, Dhillon, Ravikumar, & Sustik, 2011) using a matrix penalty term (this process is also called the adaptive lasso (Zou, 2006) determined by the SC with edges ***W*** (and *λ*_1_ = *λ*_2_):

$$\hat{\Phi }=\arg\min_{\Phi ≻0}\left(-\log\left(\det\Phi \right)+\text{tr}\left(\Theta \Phi \right)+\lambda_{1}*\sum_{j<k;W_{jk}\in SC}\left|\Phi_{jk}\right|+\lambda_{2}*\sum_{j<k;W_{jk}\notin SC}\left|\Phi_{jk}\right|\right)$$ (7)

Note that in the limiting case of *λ*_1_ = *λ*_2_, the likelihood function is the same as it is for the graphical lasso. We determine the *λ*_1_ and *λ*_2_ using cross-validation. This crucial setup simultaneously provides (1) a measure of the usefulness of the SC as a hypothesis on MEG functional connectivity and (2) serves as a principled thresholding mechanism for weak connections.

By optimizing the penalized likelihood, we leveraged the information in the SC as a hypothesis for our lasso estimate. We derive the graph *G* with vertices *V* = 1, 2, …, 114 and edges *W*_*est*_ = *G*_*ij*_ = 1, *i*, *j* ∈ *V* from the precision based on the nonzero values in $\hat{\Phi }$. The final precision matrix $\tilde{\Phi }$ is estimated under the unpenalized Gaussian likelihood for the set of edges ***W***_*est*_ defined by the graphical model using the function *ggmFitHtf* (PMTK3 toolbox; Murphy & Dunham, 2008) which optimizes (unpenalized Gaussian log-likelihood):

$$\tilde{\Phi }=\text{arg}{\text{min}}_{\Phi ≻0;\left|\Phi \right|>0=G}-\left(\log\left(\det\Phi \right)+\text{tr}\left(\left(\Theta +\delta *I\right)\Phi \right)\right)$$ (8)

Since **Θ** (covariance) is usually rank deficient, we add a small value (*δ*) along the diagonal to make it full rank. We fixed *δ* as 0.001 times the maximum value along the upper triangle of the covariance.

#### Cross-validation.

We test whether the AGL produced estimates of the precision that show reduced error relative to applying the graphical lasso using cross-validation. Note that applying the graphical lasso would be equivalent to having the penalization inside and outside the SC be equal, that is, *λ*_1_ = *λ*_2_. We estimated the appropriate value for *λ*_1_ and *λ*_2_ using cross-validation.

We split data into four ensembles, and repeated the following analysis with each ensemble. We estimated the precision $\tilde{\Phi }$_*i*_ on one ensemble of the data (*i*) and estimated the deviance when using this precision as the inverse for the covariance **Θ***_j_* for all the other ensembles *j* of the data (and vice versa). Deviance was estimated as:

$$Dev=\sum_{i=1:4}\sum_{j=1:4;j\neq i}\left(-\log\left(\det{\tilde{\Phi }}_{i}\right)+\text{tr}\left(\Theta_{j}{\tilde{\Phi }}_{i}\right)\right)$$ (9)

#### Partial coherence.

In every frequency band, or for each iteration of our simulation, we estimated the precision for complex-valued data incorporating amplitude and phase for a frequency band. The normalization of the precision (Φ) yields the partial coherence (*PC*) (Dahlhaus, 2000), estimated using:

$${PC}_{z_{1}z_{2}}={\left|\frac{\Phi_{z_{1}z_{2}}}{\sqrt{\Phi_{z_{1}z_{1}}*\Phi_{z_{2}z_{2}}}}\right|}^{2}$$ (10)

### Contemporary Methods for Functional Connectivity

We considered three alternative methods to compare the partial coherence model estimated from AGL: coherence, imaginary coherence, and the partial coherence estimated when regularizing using the L2 norm. We estimate coherence *C* from the cross-spectral density Θ, where *z*_1_, *z*_2_ are the amplitude and phase information in one frequency band from two sources, as:

$$C_{z_{1}z_{2}}={\left|\frac{\Theta_{z_{1}z_{2}}}{\sqrt{\Theta_{z_{1}z_{1}}*\Theta_{z_{2}z_{2}}}}\right|}^{2}$$ (11)

Imaginary coherence is believed to reduce the influence of volume conduction and zero phase lag connectivity (such as would exist from source leakage). The idea is to minimize this effect by estimating the consistency of the imaginary part of the cross-spectral density between two sources. We measure it using (where *imag* refers to the imaginary component of the complex value from the cross-spectral density):

$${IC}_{z_{1}z_{2}}={\left|\frac{\text{imag}\left(\Theta_{z_{1}z_{2}}\right)}{\sqrt{\Theta_{z_{1}z_{1}}*\Theta_{z_{2}z_{2}}}}\right|}^{2}$$ (12)

Coherence and imaginary coherence networks are defined using a threshold derived using bootstrapping (Zoubir & Boashash, 1998). We define a population distribution by resampling 1,000 times with replacement. We kept *C* or *IC* edges with distributions that did not cover 0 at an alpha value of 0.05.

Finally, we consider an alternative regularization to estimate the partial coherence—an L2-norm penalization. This style of regularization does not force precision values to zero but instead minimizes them to optimize the likelihood. The penalized likelihood for the L2 norm inverse is:

$$\hat{\Phi }=\arg\min_{\Phi ≻0}\left(-\log\left(\det\Phi \right)+\text{tr}\left(\Theta \Phi \right)+\eta *\sum_{j<k}{\left|\Phi_{jk}\right|}^{2}\right)$$ (13)

We need to identify a threshold for inference on the edges of the precision. Using a novel cross-validation procedure that mirrors the approach we applied under the AGL (using the likelihood function to estimate deviance), we optimize for the L2-norm penalization (*η*) and the threshold. The threshold to be applied is determined as a percentile—between 5 to 95—of the weights whose optimal value is identified using cross-validation.

### Simulations

#### Overview.

We wished to test the accuracy of the AGL to estimate the precision matrix. To do so we simulate from a generative model and attempt to recover the parameters. The generative model we use is a complex-valued multivariate normal where the nonzero values in the precision define an undirected network (as specified in Equation 4). For each simulation, and each iteration, we generated new networks with random weights for edges. While the edge locations are kept consistent within a simulation, we randomized the weights on the edges. The internal variability of each area/node changes across simulation iterations therefore changing the signal-to-noise ratio for each edge. We examined each simulation under two (or more) sampling scenarios—one where the number of samples is comparable to the number of nodes and one where there are many more samples than the number of nodes. For each simulation, where we always possess ground truth information, we assessed whether the AGL inferred (1) the usefulness of the network constraint, (2) recovered the true edges, (3) controlled the false positives, and (4) correctly estimated the edge weights of the partial coherence.

#### Simulation 1: Structural connectome simulation.

In all three simulations, to generate novel precision matrices, we retained the edge locations from the original SC but simulated random weights for the edges sampled from a normal distribution, *N*(100, 30). Finally, each edge is assigned a random phase (*μ*) based on sampling from a Gaussian distribution (mean = $\frac{\pi }{2}$, *SD* = 0.25). After multiplying each edge weight with the phase, we can generate the precision. This represents the complex-valued, circularly symmetric precision matrix (**Φ**) for a frequency band. We tested whether the precision is positive-definite by attempting to generate the Cholesky factorization of the matrix using the MATLAB function *chol*. If not, we continuously added the summed absolute value of the rows to the diagonal until the matrix was positive-definite.

Using the precision, we determined the cross-spectral density as its inverse (**Θ** = **Φ**^−1^). The cross-spectral density has a real-valued equivalent representation (Schreier & Scharf, 2010). We can treat the real and imaginary components of the CSD as separate variables governed by a joint covariance structure. Complex-valued Gaussian values were sampled using the MATLAB function *mvnrnd* operating on the real-valued CSD.

#### Simulation 2: Fake network constraint.

In the second simulation we examined whether the AGL permits inference about the hypothesized network, that is, can we use the penalizations chosen under cross-validation to judge accuracy of the hypothesized network. We began with the same approach as in the first simulation, generating precision matrices and samples from the true structural connectome. However, we changed how we applied the AGL. Rather than use the true network, we provided a fake network generated by shuffling the nodes of the structural connectome, thus allowing us to preserve the degree distribution of the original network. We shuffle nodes using the *randperm* function in MATLAB to generate 114 integers between 1 and 114 without repetition. Every iteration of the simulation, we shuffled the nodes of the SC so that the number of edges and general connectome structure are retained while the actual node identities are altered. The penalization structure under a fake network is expected to revert to the vanilla graphical lasso, with constant penalization across the entire matrix. We collapsed results across all iterations to assess if this occurred.

#### Simulation 3: Forward solution and source localization simulation.

**Figure 2.**
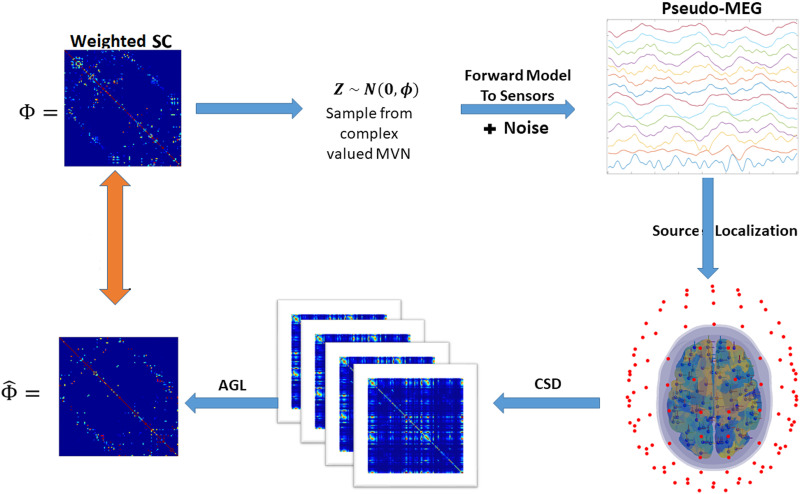
Source localization simulation, describing each figure above in a clockwise manner from top left. First, we used the SC edge locations to constrain the precision on each iteration of the simulation. We generated random weights and phases for each edge. Second, we sampled from a complex-valued multivariate normal distribution using the precision generated in the first step. Third, we used the MEG forward matrix to forward model the samples to the sensors. Fourth, we applied an inverse solution to source localize data. Fifth, we split the data into four ensembles of 120 samples (represented are the four covariance matrices from these ensembles of data). Finally, these four ensembles served as the input for the adaptive graphical lasso. The estimated precision from this procedure was compared to the original precision (orange arrow) by examining the penalizations applied, the edges recovered and edge weights recovered.

In the third simulation, we generated pseudo-MEG data. This simulation tested the ability of different methods to overcome the spatial blurring induced by the process of source localization—leakage effects and incomplete demixing of source signals. For a visual depiction of this simulation, please see Figure 2.

We first built an MEG forward model—an estimate of the magnetic field measured at MEG sensors above the scalp generated by current sources located in the brain. We built the forward model for the Neuromag MEG system consisting of 306 MEG coils at 102 locations above the scalp (shown in Figure 2). At each location, there are 3 sensors—one magnetometer that measures the component of the magnetic field passing through the coil and two planar gradiometers that measure the gradient of this magnetic field in two orthogonal directions. We made use only of the orthogonal pair of planar gradiometer coils (102 pairs of sensors at 102 locations), as planar gradiometer coils have better spatial resolution than magnetometer coils thus facilitating source localization (Malmivuo, Malmivuo, & Plonsey, 1995).

The forward model is built for a specific head model, which we developed here from the *fsaverage* MRI image from the Freesurfer toolbox (Fischl, 2012). The tessellated cortical surfaces for right and left hemisphere were extracted using the *recon-all* pipeline in Freesurfer and then downsampled to 81,000 (81k) vertices (*mris_decimate* from Freesurfer). We used this surface to constrain dipole orientation and define the volume of the model corresponding to the cortex. We generated the inner skull, outer skull, and scalp surfaces approximated with 2,562 vertices from the fsaverage head generated using the *mri_watershed* function. Using these surfaces, and with the conductivities of the scalp, CSF and brain set at 1 S/m and the skull at 0.025 S/m (i.e., 40 times lower conductivity), we applied the OpenMEEG toolbox (Gramfort, Papadopoulo, Olivi, & Clerc, 2010) to compute a boundary element model (BEM). Each row of the MEG forward matrix from the BEM is the magnetic field gradient detected across all 204 gradiometers from a unit current density source at one of the 81k cortical surface vertices.

Using the Lausanne parcellation for 114 cortical ROIs (Cammoun et al., 2012), we subdivided the cortical surface and identified vertices belonging to each ROI using the volumetric parcellation of the *fsaverage* brain. Using this organization of vertices we then reduced the representation of the current source for each ROI down to a set of three dipoles in the *x*, *y*, and *z* directions at a single location. The location of the source for each ROI was selected by taking a weighted average of vertex locations where the weight of each location was determined by the magnitude (L2 norm) of the field generated at the gradiometers. In this way, we reduced our source model to 114 source locations, with three sources at each location in the canonical *x*, *y*, and *z* directions. We computed a new MEG forward matrix (***M***) of dimension 204 × 342 using OpenMEEG which approximates the linear mixing of source activity at the gradiometers to generate the measured MEG signals.

We simulate source activity ***S*** across 114 areas using the precision with edges determined by the structural connectome, that is, one sample from the real-valued equivalent of the inverse of the precision is a 114 × 1 vector. To this source activity, we added independent noise with variance set such that the ratio of the trace of the noise to the CSD was controlled at 25 dB. We forward modeled the data to the MEG sensors. A sample of the MEG data is represented as a complex-valued vector ***B*** of length equal to the number of MEG sensors (204 sensors). The set of samples of ***B*** relates to source activity ***S*** by:

B=MS (14)

where ***M*** is the MEG forward matrix.

We localize activity to the 342 sources (three directions, along *x*, *y*, and *z* axes at 114 locations) by inverting the reduced lead field using regularized weighted minimum norm estimation (weighted L2 norm; Dale & Sereno, 1993) and applying it to data at the scalp. We estimated the inverse ***M***^−^ using (where *ν* is a penalization term):

$$\text{diag}\left(W\right)={\left\|M\right\|}_{2}^{.5}$$ (15)

$$M^{-}={\left(MW\right)}^{T}{\left({\left(MW\right)}^{T}*\left(MW\right)+\nu I\right)}^{-1}$$ (16)

We defined *ν* as the 10th percentile of the weights of ***MW***. The estimated source activity is then ***S*** = ***M***^−^ ***B***. We identify the time series for the three dipoles along the *x*, *y*, and *z* directions. Using a singular value decomposition at each ROI, we identified the optimal orientation of the dipole as the first singular vector. Using the first singular vector at each ROI, we reduced the source data from 342 × 1 to 114 × 1 for each sample. We used the source localized data as the input to the AGL to estimate partial coherence. We also estimated the coherence, imaginary coherence, and partial coherence under the L2 norm.

#### Metrics for the accuracy of the functional connectivity estimates.

Across all simulations we used the ground truth to help us understand the performance of different algorithms. To understand whether the AGL is better than the vanilla graphical lasso, we examined the penalization applied on the edges and nonedges of the network provided as a constraint in simulations 1, 2, and 3. Across all methods in simulations 1 and 3, we looked at the number of true edges recovered, the number of false positives estimated, and the accuracy of estimated edge weights. To ascertain the accuracy of estimated edge weights, we calculated the Pearson correlation between the Fisher *r*-to-*z* transformed edge weights across the set of true edges, that is, all edges in the ground truth model.

### Application to MEG Data

#### MEG data.

The MEG data we analyzed was shared by the Cambridge Centre for Ageing and Neuroscience (CamCAN). CamCAN funding was provided by the UK Biotechnology and Biological Sciences Research Council (grant number BB/H008217/1), together with support from the UK Medical Research Council and University of Cambridge, UK. This data was obtained from the CamCAN repository (available at https://www.mrc-cbu.cam.ac.uk/datasets/camcan/; Shafto et al., 2014; Taylor et al., 2017) and was conducted in accordance with the Helsinki declaration and approved by the Cambridgeshire 2 Research Ethics Committee (reference: 10/H0308/50).

MEG data was collected using a 306 sensor VectorView MEG system (Electa Neuromag, Helsinki). The 306 sensors consisted of 102 magnetometers and 204 planar gradiometers. The data were sampled at 1000 Hz and highpass filtered at 0.3 Hz. This data was run through temporal signal space separation (tSSS; Taulu et al., 2005; MaxFilter 2.2, Elekta Neuromag Oy, Helsinki, Finland) to remove noise from external sources and to help correct for head movements (location of the head was continuously estimated using Head Position Indicator coils). MaxFilter was also used to remove the 50 Hz line noise and also to automatically detect and reconstruct noisy channels.

#### Spectral analysis.

We extracted 480 seconds of resting-state gradiometer data for a single individual. We first applied a band-pass filter between 0.5 and 100 Hz and a notch filter at 50 Hz to remove line noise. We 97 built elliptic filters (designed using *fdesign.bandpass* function in MATLAB) with stop band set to 0.5 Hz below and above pass band, stopband attenuation set to 100 dB, and passband ripple set to 0.02. Band-pass filtering was then done using the *filtfilthd* function in MATLAB to minimize phase distortion. We analyzed five frequency bands: delta (1–3 Hz), theta (4–7 Hz), alpha (8–13 Hz), beta (14–29 Hz), and gamma (30–80 Hz). Within each band we optimized the dipole orientation across 114 ROIs as described in the section describing Simulation 3. Using the band-pass filtered data we were able to estimate adaptively source localized data and within each frequency band. Source localized broadband data, using band-specific source dipole orientations, was multitapered and Fourier transformed in 1-second windows. We used the all frequencies in every band, to avoid averaging over frequencies, to generate a 480 × 114 complex-valued matrix used for estimating the cross-spectral density.

Using the complex-valued data within each frequency band, we have a 480 × 114 matrix which served as the input for estimating the partial coherence. We split the 480 samples from 114 sources into four continuous ensembles of 120 samples each based on the expectation that we would have robust, stationary networks estimable with 120 seconds (Chu et al., 2012). Further, having four ensembles allowed for four-fold cross-validation. Within each ensemble we estimated the cross-spectral density and, using the AGL, the precision. We then followed the same procedure as described earlier in the section on cross-validation. Thus, we had at the end of the analysis for each subject, partial coherence across all five frequency bands.

## RESULTS

### Simple Five Node Network

**Figure 3.**
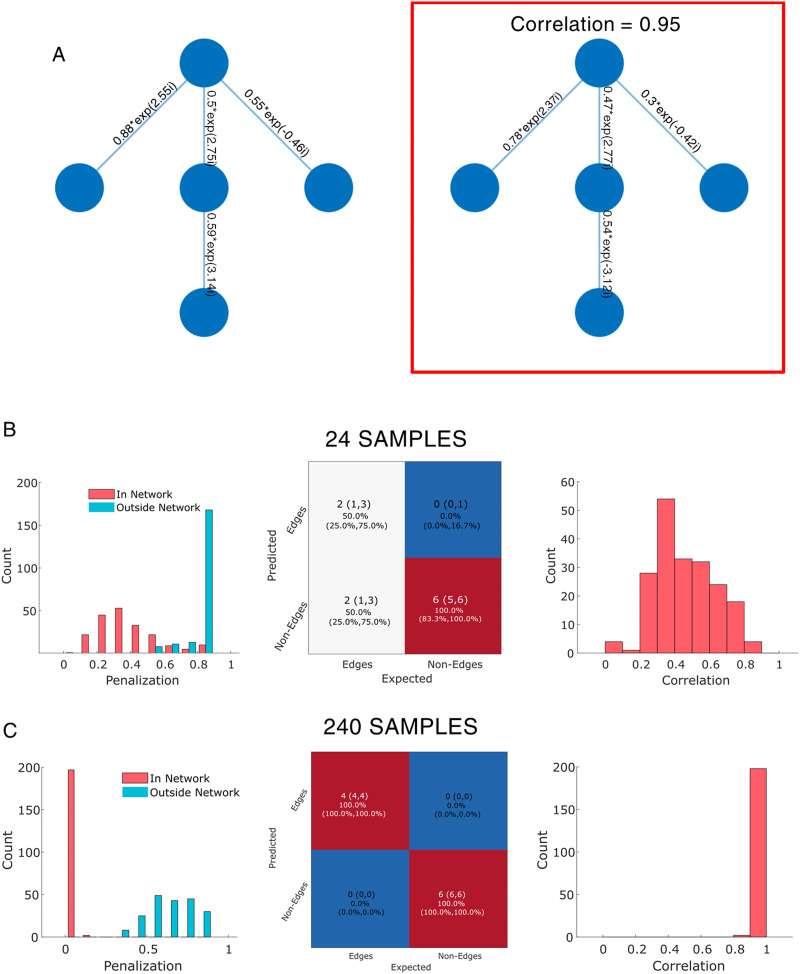
AGL provides accurate five node complex-valued network inference and controls false positives. (A) (Left) We simulate from a simple five node network with complex-valued edges. (Right) An example of a recovered network with accurately reconstructed weights when using 240 samples. (B, C) Left column shows penalization distributions for the constraint network provided (red) and outside the constraint network (blue) when using 24 and 240 samples across 200 simulations. *Y*-axis shows count of simulations and *X*-axis the penalization multiplier applied. Middle column shows confusion matrices with median (25th, 75th quartiles). Right column shows the correlation of edge weights across all true edges in reconstructed networks.

As a proof-of-concept simulation, we examined network recovery of a sparse five node network with five edges (see Figure 3A) representing the precision. We sampled data for each node from the inverse of the precision, the cross-spectral density. We apply the AGL to the observed data to extract the partial coherence; a network with weighted edges. We considered two cases, one where we have a small number of samples (24 independent samples) and, second, when we have a large number of samples (240 independent samples). Each simulation (24 and 240 samples) is repeated 200 times.

The cross-validation process allows the AGL to place the same penalization everywhere, thus the penalization values assess the usefulness of a network constraint. We see from the penalization distribution (Figure 3) that there is reduced penalization for true edges relative to nonedges, as we expected, both with 24 and 240 samples. The second metric of interest is edge recovery. In Figure 3B (middle column) we can see that the false positives are well controlled (with the distribution concentrated at zero edges) while we recover between two to all five of the true edges present despite only 24 available samples. With 240 samples (Figure 3C, middle column), we recovered all true edges in all 200 simulations and avoided any false positives in 95% of simulations. The final test is the recovery of the actual edge weights—the complex values representing connection strength and relative phase. We estimate this correspondence using a correlation between the true edges and the recovered edges. A high correlation implies that the complex-valued vectors tend to align with the orientations and strengths of the original complex-valued vectors and a correlation close to 0 indicates incorrect weight and orientation (an orthogonal vector or a zero vector). From Figures 3B and 3C we can see that correlation is 0.5 with 24 samples while it is nearly 1 with 240 samples. We conclude that we are able to recover the weights and edges of the precision even when we have only 24 samples, but with (an order of magnitude) more samples, we are almost able to recover the precision perfectly.

### Recovering the Structural Connectome

In the second simulation, we considered an order of magnitude increase in the number of nodes and edges. We used the structural connectome across 114 areas. The network is sparse, with 720 weighted edges out of a total possible of 6,441 edges. The inverse of the precision determined from the SC could represent the cross-spectral density estimated from intracranial electrocorticography (ECoG). Similar to the first simulation, we examine the performance of the AGL to estimate the correct partial coherence when we have 480, 960, 1,440, 1,920 and 2,400 samples. Since we simulate from a covariance structure with nonzero intra-ROI variance, the signal-to-noise ratio of each individual edge is modulated in every simulation iteration.

**Figure 4.**
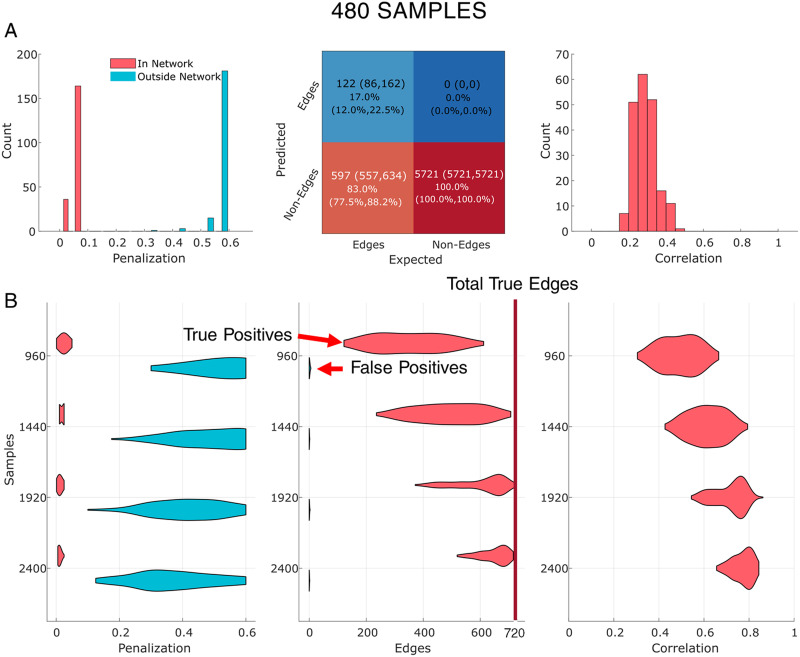
AGL recovers the partial coherence derived from the structural connectome with 114 nodes. (A) We ran 200 iterations of a simulation where we sampled from the weighted structural connectome as the precision. The left column shows the distribution of penalization on the structural connectome, and outside the structural connectome when we use 480 samples. Middle column shows the confusion matrix of edges recovered. Right column shows the correlation of all true edges with edges recovered. (B) We show changes in performance as a function of the sample size. Distributions are across all simulations. The left and right columns are similar to panel A, while the middle column now shows only the distributions of false and true positive edges recovered across 200 simulations. As the number of samples increases, AGL improves significantly, with network recovery almost perfect at 2,400 samples. In the middle column, the red vertical line represents the total number of true edges present (720).

When simulating data from the structural connectome in a low samples case (480 samples), AGL identifies the correct penalty structure (Figure 4A, left column) and controls false positives (Figure 4A, middle column). Network recovery under AGL in a high sampling situation (2,400 samples) is nearly perfect (Figure 4B). The penalization structure consistently (across all sampling scenarios) indicated lower penalization on SC edges relative to non-SC edges, the false positives are controlled (also across all sampling scenarios) and real edges identified (≥500 of 720) and finally, the edge weights, that is, the partial coherence, are well recovered (correlation ≥ 0.7). This showed that the AGL is able to infer a penalization structure that uses the structural connectome. Even when we simulated only 480 samples the AGL minimized false positives, showed the usefulness of knowledge of the SC and reasonably recovered the network weights. We conclude that low numbers of samples do not pose an impossible hurdle in judging the usefulness of the structural connectome, recovering the structural connectome and controlling false positives.

### Inferring an Inaccurate Structural Connectome Constraint

We forced model misspecification onto the AGL and examined the results. Model misspecification is done by altering the constraint provided to the AGL relative to the generative network model. We expect that the penalization structure of the AGL will reflect when we have used an incorrect network as a potential constraint: a shift in penalization toward the graphical lasso, that is, a uniform penalization. An alternative hypothesis is that the AGL always uses any constraint provided: the penalization can never approach the graphical lasso. We test these hypotheses by shuffling node identities for the SC network constraint provided to the AGL. However, we generate data from the structural connectome determined precision.

**Figure 5.**
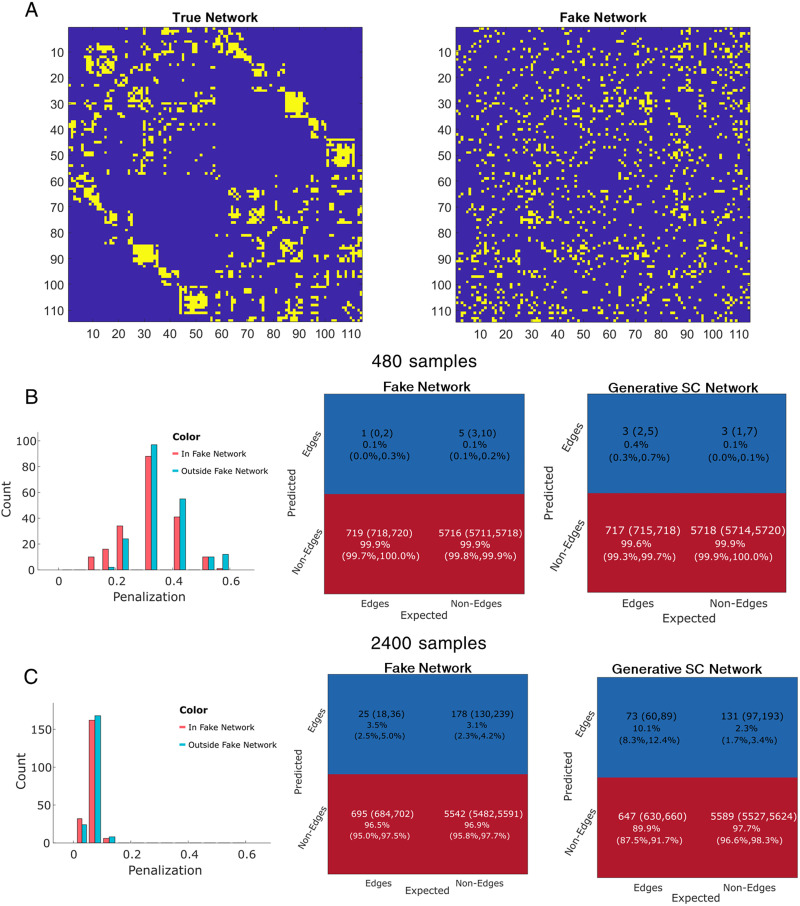
Using a fake network constraint provides no useful information. (A) On the left is the true network from which the data is simulated. On the right we show an example fake network constraint where we shuffle nodes in the true network, thus retaining the degree distribution of the original network. (B and C) The left column shows the penalization applied inside and outside the fake network constraint. Middle column shows the confusion matrix when predicting the fake network edges. Right column shows the confusion matrix when predicting the original network edges. Note that when we have 2,400 samples, more true edges from the original network are estimated and very few in the fake network.

Examining the penalization structure (Figure 5B and 5C, left column), we find that the AGL does not place lower penalization values on the fake network edges, instead approaching the flat penalization of the graphical lasso. However, this does not imply network recovery in either the fake or the true networks (Figure 5B and 5C, middle and right column), with both the false positives and the true edges suppressed in both networks at 480 samples. Penalization at 480 samples is placed uniformly high across the whole network (on and off the fake network edges). However, at 2,400 samples, the AGL places a small uniform penalization across the whole network allowing more true edges to be estimated (Figure 5C, right column), while the false positives driven by the fake network continue to be controlled (Figure 5C, middle). This suggests that while the AGL remained constrained by the network provided, (1) an incorrect network constraint can be inferred from the penalization structure and (2) with sufficient samples the true network can be partially recovered despite an incorrect constraint.

### Comparing AGL to Contemporary Network Recovery Approaches

**Figure 6.**
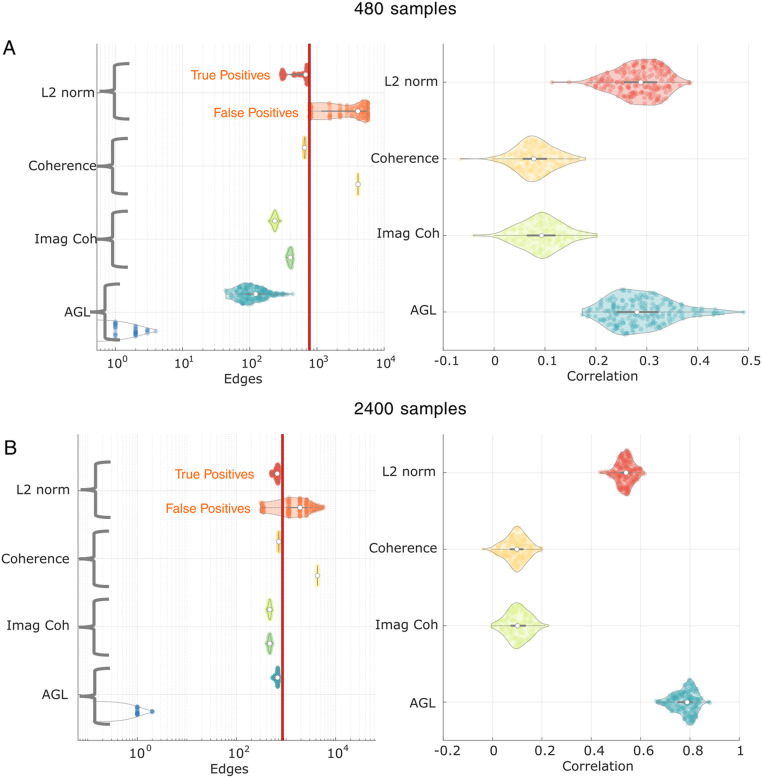
AGL outperforms alternative methods at SC recovery. We compare AGL to an L2 norm inverse, coherence, and imaginary coherence. We show two cases: 480 (A) and 2,400 samples (B). (A) (Left) For each method on the *y*-axis, there are two violin plots with the upper violin representing edges discovered in the SC (true positives) and the lower violin showing edges discovered outside the SC (false positives). No method controls the false positives as well as the AGL (see bottom violin); however, all alternative methods recover more edges in the SC. In the right column we show correlation of all true edges with edges estimated. The L2 norm inverse recovers edge weights comparably to the AGL. (B) At 2,400 samples, the AGL outperforms all other algorithms at controlling both false positives and at edge weight recovery. Note that since the L2 norm is thresholded based on percentiles of edge weights, the number of edges recovered outside the SC is discrete.

Many contemporary algorithms aim to estimate the networks scaffolding EEG/MEG/ECoG data. We compared three methods that make similar assumptions about the data as using the AGL-estimated partial coherence : coherence (Bendat & Piersol, 2011), imaginary coherence (Nolte et al., 2004), and the partial coherence estimated under an L2-norm inverse (Colclough et al., 2016; Ter Wal et al., 2018). We first compared these methods when recovering a network with structural connectome edges using 480 samples. We compared the methods on the true positives, false positives, and the network weight recovery (Figure 6).

We found that, at 480 samples, all methods were able to recover the true SC edges; however, they also estimated a considerable number of false positive edges. The AGL was considerably better at controlling false positive edges than all other methods, with the imaginary coherence performing better than coherence and the L2 norm. When estimating network weights, the L2 norm inverse and the AGL did considerably better than the coherence and imaginary coherence. At 2,400 samples, we saw similar performance differences across methods, with the AGL continuing to outperform all other methods at controlling false positives. Further, the AGL is better than the L2 norm inverse at estimating the network weights of the true network at 2,400 samples. We conclude that the AGL-estimated partial coherence outperforms contemporary algorithms at recovering the underlying network, both when we have limited independent samples and when we have large numbers of independent samples.

### Network Recovery After Source Localization

In M/EEG research, we must both recover the network from limited samples and reduce the impact of signal leakage from source localization. We apply a commonly used source localization technique (weighted L2 norm inverse; see details in Methods, section on Simulation 3) to attempt to recover sources. We apply the AGL and other algorithms to this recovered source activity.

**Figure 7.**
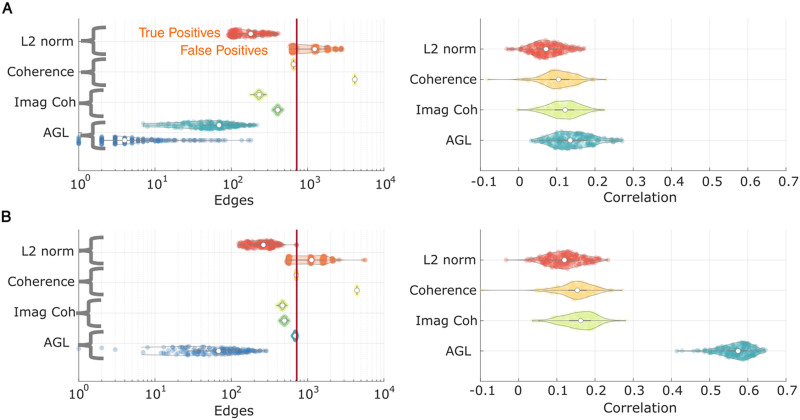
AGL outperforms alternative methods at network recovery under source localization. We generate data from a network in the brain with SC edges and forward model to MEG sensors. After applying source localization (weighted L2 norm inverse), we attempt to recover the original network. (A and B) In panel A we show results when using 480 samples and in panel B we show the results when using 2,400 samples. Left column shows the true and false positives discovered by each method. Right column shows the correlation of the true edges with estimated edges. AGL outperforms all algorithms at 2,400 samples at recovering true edges, controlling false positives and recovering edge weights.

Examining the 480 sample case (Figure 7A), we see that the AGL continues to outperform all other methods at controlling false positives in the network. However, other network recovery techniques were comparable in recovering true SC edges, with the coherence recovering all edges but also including a large number of false positives. All methods were comparably poor at recovering the network weights. When we have more samples (2,400; see Figure 7B), we see that the AGL clearly outperforms all algorithms in all metrics measured, with the correlation with network weights reaching 0.58. This suggests that when more samples were available we were able to partially overcome the difficulties imposed by source localization by using the AGL-estimated partial coherence.

### Application to MEG Data

We extracted 480 seconds of preprocessed resting-state MEG data from a single subject from the open-source CamCAN dataset. We source localized this data (using weighted L2 norm; Dale & Sereno, 1993) to the 114 areas of the Lausanne parcellation. After source localization, we used 1-second windows to get amplitude and phase samples at each frequency from 1 to 50 Hz using the multitaper method. We applied the AGL with our cross-validation procedure (see section Cross Validation) to estimate the partial coherence. Note that since we examined only a single subject, we intended this to only be a demonstration of how the AGL-estimated partial coherence could be used. Further, we do not have a ground truth in this situation so we focus on the penalization structure to infer if the structural connectome (SC) is useful information in modeling the coherence. We did find that the SC serves as a useful constraint in the delta (2/3 frequencies), theta (1/4 frequencies), and beta bands (11/15 frequencies), but not in the alpha (0/6 frequencies) or gamma (0/20 frequencies) bands. The null results in the alpha and gamma band indicate that the measured functional connectivity involves other connections, for example, thalamocortical or other subcortical projections not included in the structural connectome. Finally, when we applied a fake network—a shuffled SC—as a potential constraint, we found that none of the frequency bands use the constraint applied and the algorithm chose to use vanilla graphical lasso. This indicates that only the SC serves as a useful constraint in the delta, theta, and beta bands.

**Figure 8.**
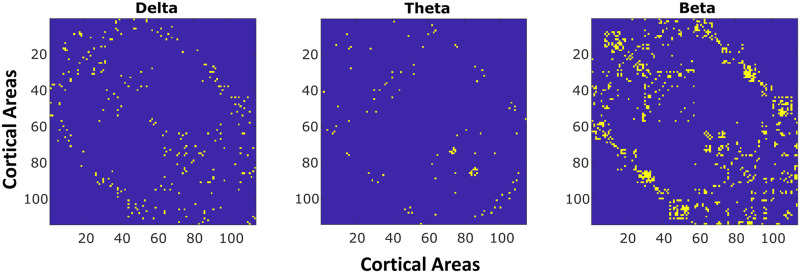
AGL recovers unique partial coherence networks in different frequency bands. We apply the AGL to delta, alpha, theta, beta, and gamma frequency bands. We show here results from the bands where there was a lower penalization applied to the SC. In the delta, theta, and beta bands we show the edges that were maximum in strength for that band. This selection shows how there exist different networks at each frequency with the beta band showing the greatest spread across the SC and the theta band network showing the greatest selectivity.

For the cases when the SC was a useful constraint, the partial coherence estimates the edges of the SC that are relevant for each frequency, which can be a subset of the structural connectome. We examined the edge weights in the delta, theta, and beta bands, looking for which band had the highest weight at each SC edge. We show this in Figure 8. Beta band networks tend to have connections distributed across the cortex, while theta and delta connections are more circumscribed. Delta band shows connections within frontal and cingulate regions and from frontal/cingulate to parietal regions. Theta band shows consistent connectivity across left and right hemispheres between temporal and parietal/occipital regions. Beta band connectivity dominates throughout the rest of the structural connectivity, with little specificity. The relevance of the structural connectivity to beta band functional connectivity is consistent with past research (Garcés et al., 2016). We conclude that the AGL can be applied to empirical data to discover networks in different frequency bands.

## DISCUSSION

We developed a model of MEG coherence constrained by knowledge of anatomical connectivity in the structural connectome. We showed that we can accurately infer the weighted network connectivity by means of partial coherence, for the first time, using the AGL. This method can assess if the structural connectome is useful as a constraint for estimation of the partial coherence by comparing the penalization applied to the structural connectome to the penalization applied outside it. Finally, we demonstrated how, when the functional connectivity is simulated from the structural connectome, the AGL-estimated partial coherence outperforms coherence, imaginary coherence, and the L2-norm estimated partial coherence.

### Functional Connectivity Network Using Partial Coherence

The AGL yields a new measure of functional connectivity that is based on the expectation that the structural connectivity scaffolds the functional connectivity. Critically, the method also allows functional connections to exist that are not prescribed in the structural connectome. Given the ability of the partial coherence to reduce false positives and provide an accurate definition of a path (Avena-Koenigsberger et al., 2018), it serves as a useful electrophysiological functional connectivity measure for network analyses (Reid et al., 2019). Further, the precision can potentially be applied toward other analyses that attempt to decode the causal direction of connections (Baccalá & Sameshima, 2001; Sanchez-Romero & Cole, 2021).

### Estimating Partial Coherence Using the Structural Connectome

Regularization is essential to estimate partial coherence for large networks. We argued that an L1 norm regularization is more intuitive than the L2 norm because the structural connectome is sparse. We can explicitly incorporate the structural connectome (SC) into the partial coherence estimate through the AGL. Past work applying a matrix penalty term to the graphical lasso (Pineda-Pardo et al., 2014)—using it to estimate the partial correlation—has directly forced the SC connection weights onto the penalization weighting. In contrast to Pineda-Pardo et al. (2014), we expected that the SC strengths are unlikely to map directly onto the strengths of the precision due to individual differences and variance within individuals across functional brain states. In addition, we expected that the SC can have different contributions across frequency bands yielding different connection weights. For these reasons we used the binarized SC to potentially organize the L1 penalization, that is, we allowed the penalization to entirely ignore the SC if appropriate.

### Using Larger Numbers of Samples in Functional Connectivity Research

We found that the accuracy of network recovery is contingent on the number of samples used. While a subset of the network was recoverable when samples were comparable to nodes, from simulations it appeared that there was considerably improved performance with higher numbers of samples. While past work has suggested that for coherence there can be convergence within a few hundreds of samples (Chu et al., 2012), we saw that for the imaginary coherence and for partial coherence, larger numbers of samples significantly improved performance. This knowledge provides impetus to use longer recordings (10 minutes or more) to estimate resting-state electrophysiological functional connectivity, similar to recent work in functional-MRI research (Gordon et al., 2017).

### Limitations

In the simulations, we assumed a generative model where brain areas show random oscillatory behavior linked by the structural connectome. This could be represented using a zero-mean complex multivariate normal with a circularly symmetric precision. More detailed mean-field models of neural activity may be more phenomenologically accurate (Cabral et al., 2014); although, past work suggests there is limited gain in using them when explaining empirical data (Finger et al., 2016; Messé, Rudrauf, Giron, & Marrelec, 2015). As such, there is value in having multiple models to explain the data as a function of the hypothesis being tested.

While partial coherence offers a clearer definition of a direct connection between areas, it is potentially susceptible to false positives, depending on the nature of causal direction of common effects in the network (Sanchez-Romero & Cole, 2021). Specifically, if two nodes *A* and *B* are directly influencing a third node *C*, and *A* and *B* are unassociated, then a false positive connection can appear between *A* and *B*. As such, the partial coherence potentially could be better used in concert with coherence as proposed by Sanchez-Romero and Cole (2021).

In humans, structural connectivity is only estimated from diffusion weighted imaging and is an imperfect measure, subject to its own limitations (Karnath, Sperber, & Rorden, 2018; Maier-Hein et al., 2017). There are difficulties in tractography linked to overlapping fiber bundles that make it hard to identify correct bundle endpoints, and strict correction of incorrect streamlines can rapidly lead to large numbers of false negatives (Maier-Hein et al., 2017). The decision to remove nonhomologous interhemispheric connectivity may also have introduced a few false negatives. Finally, we used a group-averaged SC template for all the subjects, and while individual variability in SC is low (Chamberland et al., 2017), better models may be built using an individualized SC estimate. An important future direction will be to examine the optimal structural connectivity estimate for MEG data.

Source localization can be formulated in several ways based on prior assumptions. While we used a weighted *L*2 norm inverse, beamformer reconstruction approaches are also quite common in MEG (Brookes et al., 2011; Gross et al., 2001) and require investigation within this framework. Bayesian techniques accounting for priors more explicitly can afford better source reconstruction (Baillet et al., 2001; Wipf & Nagarajan, 2009). Examining these alternative approaches was beyond our scope, but the AGL is equally applicable under these alternatives. Additionally, we chose to limit our analysis to an SC with 114 nodes; a future extension to this work might examine cases with more (or fewer) sources. We also ignore for our purposes subcortical source activity and connectivity. This may have led to the large variation in the estimated results in the MEG data. In this example, the alpha and gamma rhythms may not have mapped onto the structural connectome because of strong thalamacortical contributions. Estimation of subcortical activity in MEG, while possible, is difficult without explicit prior knowledge (Krishnaswamy et al., 2017), and would also potentially benefit from including the magnetometer recordings and developing individual subject head models.

### Conclusion

Understanding the relevance of different potential constraints, such as source modeling and the structural connectome, on a ”big data” measurement technique such as MEG data improves our ability to infer genuine signal variability from noise. This work developed a simple model derived from the constraint of the structural connectome and demonstrated that we can recover the model parameters in simulations. This method is useful in clinical situations and in cognitive neuroscience for understanding network structure. For example, estimating a gamma band partial coherence network in a working memory task to understand which structural connections are most strongly activated. Another example, as we have recently demonstrated in fMRI research (Wodeyar et al., 2020), is to examine the influence of lesions and concomitant structural disconnection on MEG or EEG functional connectivity. Interpreting M/EEG coherence is contingent on building and comparing different models of the data, and we believe our work takes us a significant step in this direction.

## SUPPORTING INFORMATION

Supporting information for this article is available at https://github.com/wodeyara/AdaptiveGraphicalLassoforParCoh (Wodeyar, 2022).

## AUTHOR CONTRIBUTIONS

Anirudh Wodeyar: Conceptualization; Formal analysis; Investigation; Methodology; Software; Visualization; Writing – original draft; Writing – review & editing. Ramesh Srinivasan: Conceptualization; Funding acquisition; Methodology; Resources; Supervision; Writing – review & editing.

## TECHNICAL TERMS

Leakage effects: Artifactual shared activity across brain regions after M/EEG source localization.
Partial coherence: Transformation of the cross-spectral density that allows inference of conditional dependencies.
L2-norm penalization: Penalizing the sum of squares of precision weights, which we normalize to get the partial coherence.
Penalization structure: The L1-norm penalties chosen for the edges and non-edges of the network constraint provided to the adaptive graphical lasso.
L1-norm penalization: Estimating the precision while penalizing the sum of the absolute values of the precision.
Adaptive graphical lasso: Extension of Ll-norm penalization that permits different penalties to be applied to different edges of the precision.
Deviance: Estimate of the goodness-of-fit between the estimated precision and out-of-sample estimated cross-spectral density.
Forward model: Estimate of the mixing of the magnetic field at the MEG sensors from brain source of activity.
Mean-field models: Mathematical model of the mean neural population dynamics.

## Supporting Information

https://github.com/wodeyara/AdaptiveGraphicalLassoforParCoh
